# Accelerating systems thinking in health: Perspectives from the region of the Americas

**DOI:** 10.3389/fpubh.2023.968357

**Published:** 2023-03-16

**Authors:** Saenz Rocio, Echandi María Paulina, Rojas Karol, Solís Luis Fernando, Gómez Ingrid

**Affiliations:** ^1^Center for Research in Nursing and Health Care, University of Costa Rica, San José, Costa Rica; ^2^Independent Researcher and Professional Consultant, Boston, MA, United States; ^3^School of Public Health, University of Costa Rica, San José, Costa Rica

**Keywords:** systems thinking, stakeholder mapping, health system, Region of the Americas, SYSTAC, community building

## Abstract

**Introduction:**

The Systems Thinking Accelerator (SYSTAC) is a community to engage, connect and collaborate to elevate the field of systems thinking with a focus on low- and middle-income countries, highlighting the need to identify existing capacities within research and at the practice level. The study aimed to explore if there is a perceived need for and benefit from the application of Systems Thinking tools for analysis and diagnosis of problem-solving within Healthcare in the Region of the Americas in 2021 and the existing capabilities.

**Methods:**

The identification and deconstruction of the needs, demands, and opportunities regarding systems thinking in the Americas were approached by: (i) adapting the tools and Systems Thinking definition to reflect regional nuances, (ii) identifying stakeholder exercise, (iii) needs assessment survey distribution, (iv) stakeholder mapping analysis, (v) workshop. More information on the adaptation and execution of each tool can be found below.

**Results:**

123 stakeholders were identified, of which 40 participated in the needs assessment survey. 72% of respondents indicated little knowledge of the tools and approaches of systems thinking but a high interest in developing them, as stated by 87% of respondents. Qualitative tools were most frequently used, such as brainstorming, problem trees, and stakeholder mapping. Systems thinking is mainly used when conducting research, implementing, and evaluating projects. A clear need and want for training and developing capacities in health systems thinking were identified. However, in practice, systems thinking faces challenges like resistance to change and to the transformation of health processes, barriers at the institutional level, and other administrative disincentives that hinder its application, being institutional transparency, political will, and the articulation of the actors the main challenges.

**Discussion:**

Strengthening and building personal and institutional capacities in systems thinking theory and practice requires overcoming challenges such as lack of transparency and inter-institutional cooperation, the low political will to implement it, and difficult stakeholders' integration. As a first step, it is crucial to understand further the stakeholder network and the capacity needs of the region, gain buy-in from strategic players to establish the use of system thinking as a priority, and develop a roadmap.

## 1. Introduction: Systems thinking in the Region of the Americas

The Systems Thinking Accelerator (SYSTAC) defines Systems Thinking as an approach to problem-solving that views problems as part of a wider dynamic system and therefore requires a deeper understanding of the behavior of complex adaptive systems in designing, evaluating, and implementing health policies to maximize health and health equity (1). It recognizes and prioritizes the understanding of linkages, relationships, interactions, and interdependencies among the components of a system that give rise to the system's observed behavior. Systems thinking is a philosophical frame, and it can also be considered a method with its own tools (2). Systems thinking can be used in research, policy, or practice.

Since the publication of the Alliance for Health Policy and Systems Research (the Alliance) flagship report “Systems Thinking for Health Systems Strengthening,” systems thinking in health policy and systems research (HPSR) has been widely accepted. However, it has become apparent in recent years that systems thinking in HPSR has largely remained (i) the purview of researchers, and (ii) perceived as primarily conceptual, with limited examples of applications of systems thinking available—particularly in policymaking and practice, and especially in low- and middle-income settings (LMICs).

For this reason, the Alliance is developing the Systems Thinking Accelerator (SYSTAC) initiative, a community for systems thinkers to engage, connect and collaborate, to elevate the field of systems thinking to improve health. The Alliance is launching the SYSTAC as a global community-of-practice, with a focus on practitioners in health systems in LMICs.

A core component of SYSTAC will be to bring together a diverse group of stakeholders that goes beyond academia and research to include practitioners and decision makers. It aims to be an ecosystem of partners working to advance health. The high fragmentation of health systems in LMICs (3) forces stakeholders to work in silos with very limited integration of the different components of the health system, thus constraining their ability to adopt multi-sectoral approaches. SYSTAC aims to increase the critical mass of systems thinkers and connect those who have been working in isolation. Although SYSTAC focuses on systems thinking within the health sector, actors and expertise in systems thinking from other sectors will be welcomed to contribute and improve systems thinking approaches for health.

As part of the process to build and create a community and platform for the Region of the Americas, a road map was developed and the research team was contacted to bring together different actors from the Region of the Americas (decision makers, researchers, professionals in the field), aiming to strengthening Systems Thinking capacities and its application in Health Systems in the region, and connect them with other regional institutions. The research team is an interdisciplinary team in Costa Rica with expertise in public health and health systems research in Costa Rica and in the Region of the Americas.

As a first step for developing the SYSTAC-Region of the Americas community an initial needs assessment has been carried out, identifying the key needs, capabilities, demands and opportunities for the application of Systems Thinking in Healthcare in the Region of the Americas.

Countries in this region share many economic, political, social, and cultural similarities but at the same time vary among themselves, with diverse Health Systems and capabilities. The Region of the Americas is one of the regions with the lowest investment in public health, additionally historical characteristics of the health systems in the region have complicated effective responses to challenges in health (3).

These challenges are due in part to a fragmentation and segmentation of medical services (3) based on the poor from the formal sector, resulting in significant gaps in health care access and quality for this group. Within the formal sector, the private sector varies in extension and importance within the region, but mainly requires people to have insurance or pay-for-service. The public sector in Region of the Americas countries instead is divided into two health systems segments: a relatively well funded social security for salaried workers and their families and a Ministry of Health system serving poor and vulnerable people with low standards of quality, except for Costa Rica (4).

This fragmentation in the health system and service delivery, together with the poorly regulated private sector, becomes a challenge for efficient services. Health care performance and quality of health service delivery is also weak, with poor primary health care systems and limitations in advance hospital services, with little progress in the past years in several countries. Decentralization of funding and decision making is another common issue to the Region of the Americas. This is a process that has developed to respond to the need of promoting development in the regions and provinces within the countries. However, especially related with public health and health systems, it has generated more complicated environments for governance, different levels of wealth in the regions, differences in performance, priorities, and capacities to respond to health issues and even politicization of health decisions (4).

Taking the above into consideration, and the current health crisis due to the COVID-19 pandemic (5), it is vitally important to identify and strengthen regional health capacities in the Region of the Americas by leveraging Systems Thinking capabilities and tools, both for their application at the scientific and practice level. The focus on health by SYSTAC, rather than the “health sector,” means that it aims to reflect the reality of health, which is complex, necessitating a multi-sectoral, regional lens, and interdisciplinary collaboration for improving health.

For this reason, the goal of this research study was to explore if there is a perceived need for and benefit from the application of System Thinking, within healthcare, in the Americas Region, in the year 2021, by decision makers, researchers, and professionals in the field. As well as investigate what the existing System Thinking capabilities are within the region and field. Next steps, after this initial assessment, will include an effort to build a SYSTAC-Region of the Americas community that brings together the different key stakeholders needed to strengthen regional capacities for the application of Systems Thinking at the regions' Health Systems.

## 2. Methods

To explore the perceived needs and demands of systems thinking practitioners, researchers, managers, and decision makers in the field of healthcare in the Region of the Americas, as well as the current capabilities the following approach was undertaken:

1. Map and catalog existing and potential actors and initiatives in the Region of the Americas and further identify actors to join SYSTAC.
2. Survey the needs and demands of systems thinking practitioners, researchers, managers, and decision makers in the field of healthcare in the Region of the Americas, in order to inform a roadmap to improve the capacity to apply systems thinking in health in the region.
3. Document the key barriers and opportunities for applying systems thinking in healthcare in the Region of the Americas.

For the fulfillment of the above the first step was to identify an initial list of stakeholders to contact for participation in the needs assessment and to adapt the SYSTAC needs assessment tools to the local context and language. This included the adaptation of SYSTAC's definition of Systems Thinking to regional nuances and context. In addition, the tools/techniques adapted were: i. a needs assessment survey, ii. stakeholder prioritization exercise, iii. workshop. More information on the adaptation and execution of each of these tools can be found below. Firstly, the needs assessment survey was conducted reaching out to a wide set of stakeholders. The survey findings were used to inform the stakeholder prioritization and to plan the workshop. Findings from the survey, stakeholder prioritization and workshop were integrated as a product of the study.

### 2.1. Stakeholder identification

A stakeholder identification brainstorming session was conducted by the research team to identify actors according to the role of decision makers, health practitioners, providers, health professionals, and researchers. Decision makers are those who are most responsible for developing policies and/or making funding decisions, such as global and national policy makers and funders. Researchers are those who study a phenomenon, but are not per se involved in delivery, implementation, or decision making around that phenomenon. Health professionals are those who are engaged in service delivery and/or implementing policies, health promoters and educators. Health practitioners are healthcare providers who are directly engaged with the provision of medical care. The list of identified stakeholders was further developed to contain (i) sector, (ii) institution, and (iii) regional scope of each stakeholder. In addition, stakeholders were listed from different sectors in health such as academia, NGOs, independent providers, private and public sector. This initial stakeholder list was created mainly based on existing work networks, and identification of regional institutions linked to the research team.

### 2.2. Needs assessment survey

A needs assessment survey was developed and deployed to stakeholders identified above to gain insights on the regional capabilities, needs and interests regarding Systems Thinking.

As a first step the survey and an introduction note on SYSTAC/invitation to participate in the survey were created. The note and survey were developed collaboratively by the research team through a series of internal working sessions in which both were created, refined, and approved. The note and survey were shared with the regional stakeholders identified by the research team *via* email, and they were given 2 weeks to complete the survey. During this time, they were contacted once again directly by phone or email as a reminder.

The survey was developed using google forms and structured in three segments: (a) an initial segment to gather general information on the participants such as contact information, demographic, and occupational information, (b) a second segment to gather data on participants' knowledge and interest regarding systems thinking, its application and tools to inform the stakeholder mapping and prioritization exercise found in the next subsection. Additionally, this segment explored the challenges and opportunities faced when implementing systems thinking in the Region of the Americas, and (c) a final segment to document resources, initiatives and additional systems thinking stakeholders found in the region. The survey included both close-ended and open-ended questions, it contained a total of 23 questions (21 multiple questions, and 7 open-ended questions).

A descriptive statistical analysis, conducted in excel, was undertaken to analyze the data gathered in the survey and leverage it for the creation of the workshop.

### 2.3. Stakeholder mapping and prioritization

A description, prioritization, and classification of the survey stakeholders was conducted. Validating the following categories: sector, institution, regional scope; and then according to their levels of interest and Systems Thinking knowledge. The level of knowledge and initial opinion about applied systems thinking, their needs, demands, and capacities were identified through the needs assessment survey. Additionally, their acting role as decision-makers, practitioners, managers, and/or researchers was identified.

The stakeholder mapping exercise was conducted leveraging thereafter and aimed to refine and expand the information gathered during the initial consultation, with the main objective of guiding and the design of the SYSTAC-Region of the Americas community while ensuring SYSTAC fulfills a relevant role in the existing regional ecosystems and with the intent that no key stakeholder is forgone. Having said this, the process of identifying all key stakeholders is ongoing and does not conclude with this study. Gathering this information allowed the team to have a clearer overview of the regional actors with awareness/interest in systems thinking in the region, the relationships between stakeholders, the needs and demands for applied systems thinking, and how these can be strengthened to inform how SYSTAC will build on or complement other regional activities; and plan how the regional activities will engage with the different stakeholders.

To identify the level of knowledge/interest in systems thinking of the stakeholders, the matrix “Categories for the mapping of stakeholders according to their knowledge and interest in Systems Thinking” provided by SYSTAC Central was used and adapted (Table 1).

**Table 1:** Categories for mapping of stakeholders according to their knowledge of and interest in systems thinking.

**Target Audience**	**Definition**
High knowledge/high interest	Those who are more wellversed and knowledgeable of systems thinking tools and approaches and are interested in participating in a community
Low knowledge/high interest (I)	Those who are not aware of system thinking and wish to learn about the topic, they may have minimal knowledge or could be applying systems thinking approaches or methods without knowing it
High knowledge/Low interest (II)	Those who are more wellversed and knowledgeable of systems thinking tools and approaches but are not interested in participating in a community
Low knowledge/Low interest	Those who are not engaged in systems thinking and not interested in developing system thinking knowledge

### 2.4. Workshop

To explore the needs, barriers, and opportunities for accelerating the application of systems thinking in health in the region, and linked to the stakeholder mapping conducted, the 123 stakeholders identified initially were convened to participate in the workshop entitled **“*Accelerating Systems Thinking in Health in the Region of the*** ***Americas.”*** The workshop was held on May 27, 2021, in virtual mode through *Zoom.us* tool, using a theoretical-practical methodology based on dialogue and participation for the collective construction of knowledge, and led by the research team.

For its realization, methodologically two stages were proposed: preparation and execution.

In the Preparation Stage, there were five phases: (a) Analysis of three local successful experiences in health from a people-centered perspective and with intersectoral participation, that reflected the application of systems thinking and its tools, even though they were not strategies designed within the framework of this approach. (b) Selection of one of the successful experiences and elaboration of a case study: “*Conceptual and practical application of the Systems Thinking approach and its tools in a health initiative: Breast Cancer Patient Navigation Project in Costa Rica”* (see Supplemental material), (c) Review of the conceptual elements of Systems Thinking in Health and its tools, applied in the successful experiences identified, and how this led to change; and (d) Joint construction of a methodological proposal for the workshop that included the activities to be carried out, materials, time, and selection of facilitators within the research team, which was presented, discussed, adapted and validated in 4 working sessions of the team of researchers.

The Execution Stage was developed in four blocks: welcome, framing, workshop development through the analysis of the case study, and final reflections. The welcome activity included a presentation of the research central team and participants, followed by a contextualization of the initiative, the reason for the call and the objective of the workshop. This was followed by a discussion and validation, with the workshop participants, of the adapted definition of Systems Thinking in health proposed by the research team and applied to the Region of the Americas.

During the workshop, the analysis of the case study was carried out through an exercise in four subgroups, each one facilitated by a representative of the SYSTAC- Region of the Americas research team with the support of questions to generate dialogue. Finally, in the plenary session, each person facilitating the subgroups presented the main discussions and group dialogue to the audience. For the activity corresponding to the last block “Final Reflections,” a dynamic with the *Padlet.com* tool was proposed to individually share ideas about possible training opportunities, how to strengthen systems thinkers' networks, new linking actors and ideas to outline a regional acting route. These topics will be considered an essential starting point in the continuity of the process toward the Systems Thinkers community building as they will help to address the priorities in the region.

## 3. Results

To have a conceptual starting point, the definition provided by SYSTAC about systems thinking was used as a reference for the research team, translated to Spanish and adapted with a local lens to the region. The definition of Systems thinking proposed by the SYSTAC-Region of the Americas team, which was validated with the stakeholders during the workshop is as follows:

“A needs-solving approach that views problems as part of a larger, interdependent dynamic system and therefore requires deeper understanding. It is about understanding open systems, with adaptive, resilient, and complex behaviors, in which health policies are designed, evaluated and implemented to maximize health and equity. Recognizes and prioritizes the understanding of the links, relationships and interactions between the different components that make up the system. This is a conceptual and practical approach that, in turn, considers various methods with their own tools. Systems thinking can be used in research, policy or practice.”

### 3.1. Stakeholder identification

During the stakeholder identification brainstorming section 123 stakeholders were identified and later invited to participate in the study. Stakeholders corresponded to decision makers, health practitioners, health professionals and/or researchers within the Health System. The amount was identified by the research team as: 31 were decision-makers, 13 practitioners and 21 researchers, and 72 health professionals who develop various actions associated with health services. This list contained a preponderance of action at the national level and only 4 actors with a regional scope. The list included stakeholders in the age range of 25 to 75 years old. Finally, identified stakeholders came from universities, NGOs, hospitals, private and public health sector.

### 3.2. Needs assessment survey

From the 123 identified stakeholder invited to participate in the survey 40 answers were received, corresponding to a response rate of 34%. Respondents came from all four communities of interest highlighted by the research team. The 60% of the respondents worked at the country level, 23% of respondents worked at the regional level and 16% of respondents worked at the global level but were based out of the Region of the Americas, while only 3% of the respondents worked at the district or local level. Respondents worked mainly in Costa Rica and across different countries in America, some of the countries which their work impacts are Canada, USA, Mexico, Salvador, Guatemala, Honduras, Nicaragua, Costa Rica, Haiti, Cuba, and Ecuador (Table 2).

**Table 2:** Characterization of the stakeholders participating in the needs assessment survey.

**Category**	***n*** **(%)**
**Age**	
<25 years	1 (3%)
26 to 35 years	7 (18%)
36 to 45 years	10 (25%)
46 to 55 years	10 (25%)
56 to 65 years	6 (15%)
>65 years	6 (15%)
**Gender**	
Female	21 (53%)
Male	19 (48%)
**Respondent's classification**	
Management	18 (45%)
Researchers	13 (33%)
Decision makers	5 (13%)
Practitioners	4 (10%)
**Geographical reach of their work**	
Global to community	1 (3%)
Global	5 (13%)
Regional	9 (23%)
Country	24 (60%)
District	1 (3%)
Community	0 (0%)

**Figure 1:**
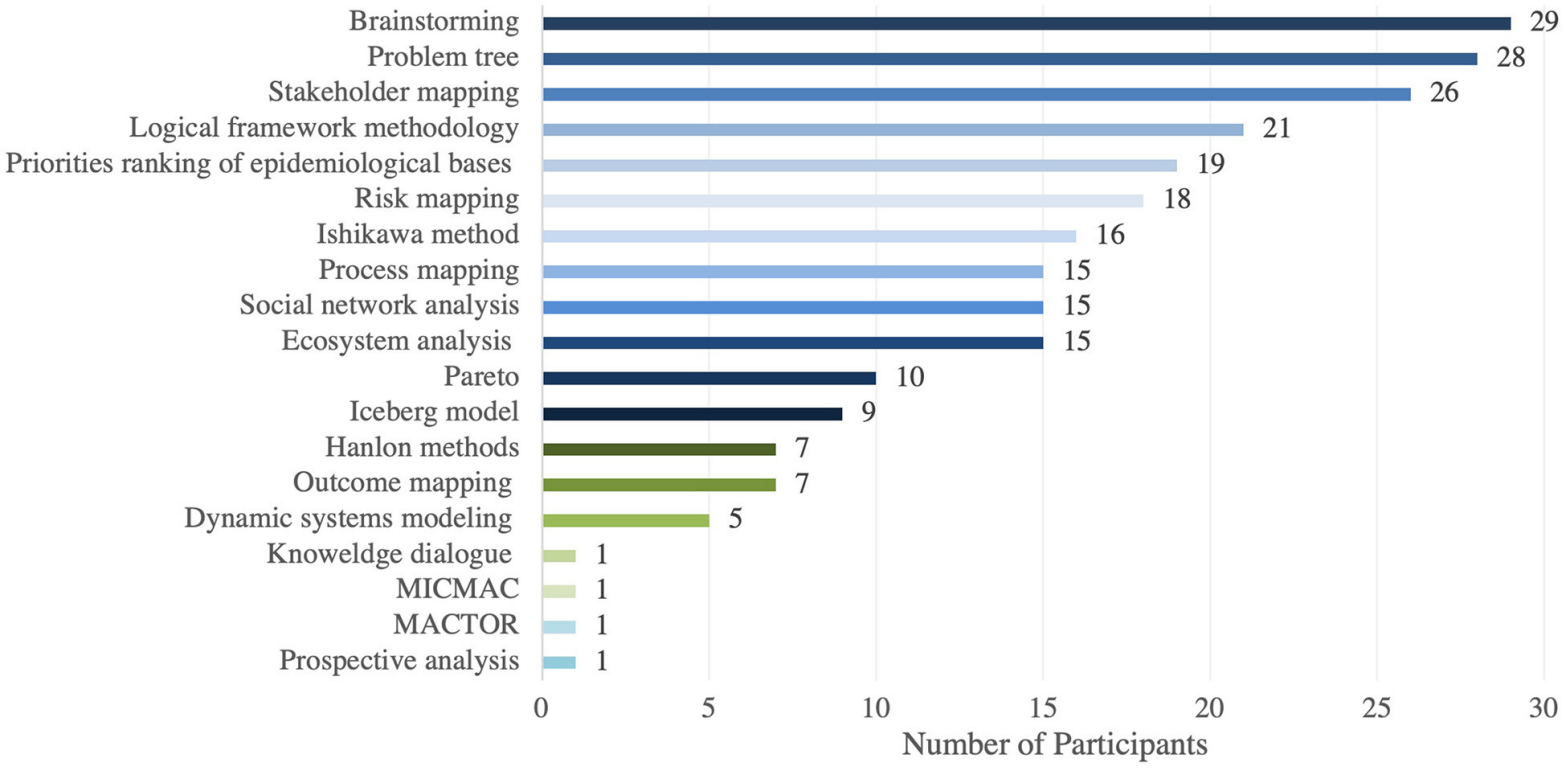
Stakeholders most commonly use systems thinking tools in the needs assessment survey.

**Figure 2:**
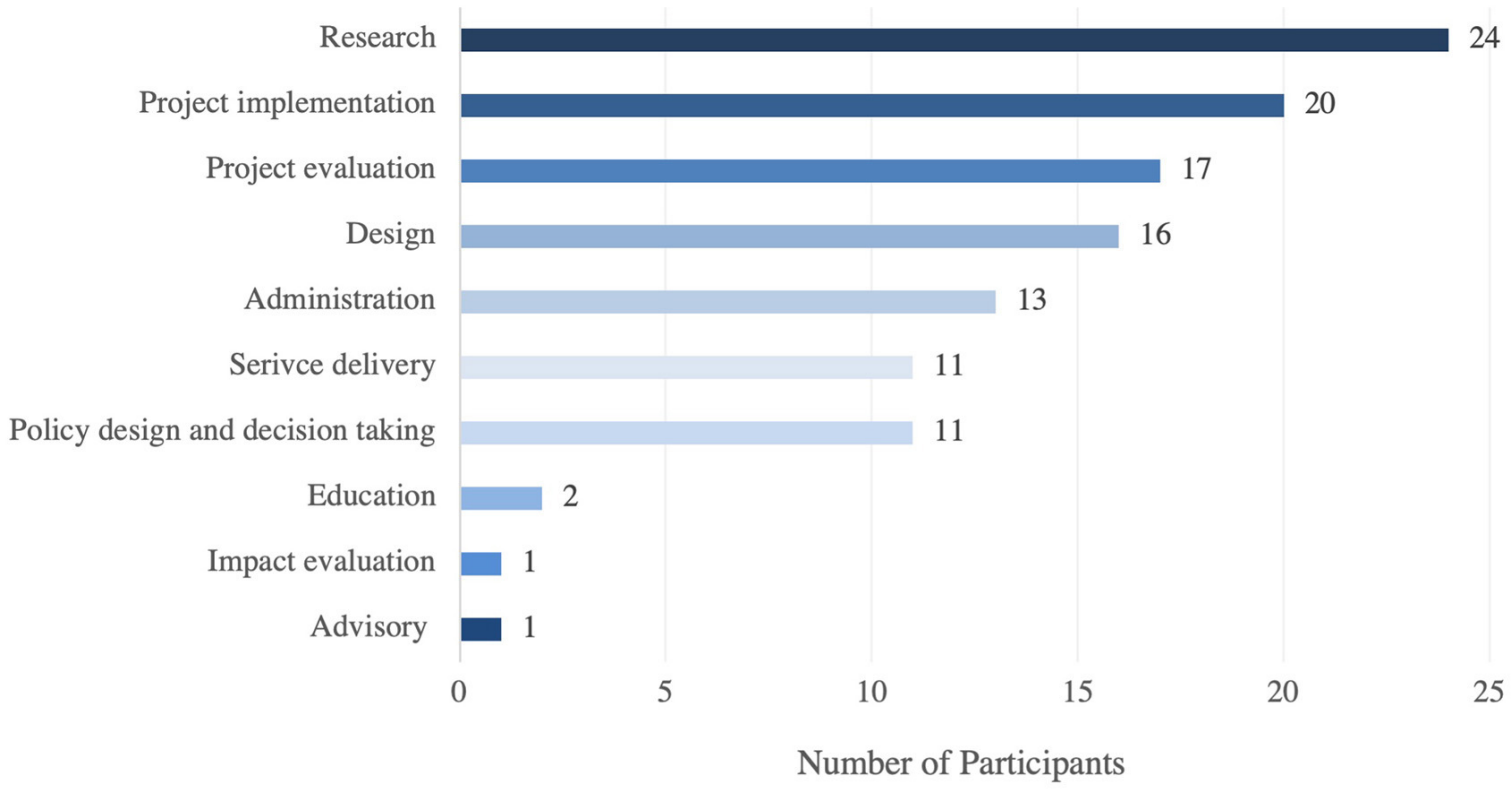
Scenarios with experience in the use of systems thinking tools.

A 75% of the participants reported having used systems thinking, 12.5% are not sure and 12.5% reported not having used this methodology before. The 12.5% of respondents who have not used Systems Thinking before were asked to skip to the last two questions of the survey to gage their interest in learning more about Systems Thinking, for this reason the denominator used for the following percentages is 35. Of the 35 respondents that have used or may have used systems thinking previously, 68.6% reported having used systems thinking tools and 31.4% are not sure that they have used the tools. Having said this, when the 35 participants were asked to select from a list of systems thinking tools (e.g., problem tree, process mapping, brainstorming, network analysis, etc.) that they have used, all of them selected one or more tools. The tools most frequently used were qualitative tools such as brainstorming, used by 83% of the respondents, problem trees used by 80% and stakeholder mapping used by 74% (Figure 1). A 69% of survey participants use Systems Thinking when conducting research, 57% when implementing projects and 49% when evaluating projects, as seen in Figure 2.

**Figure 3:**
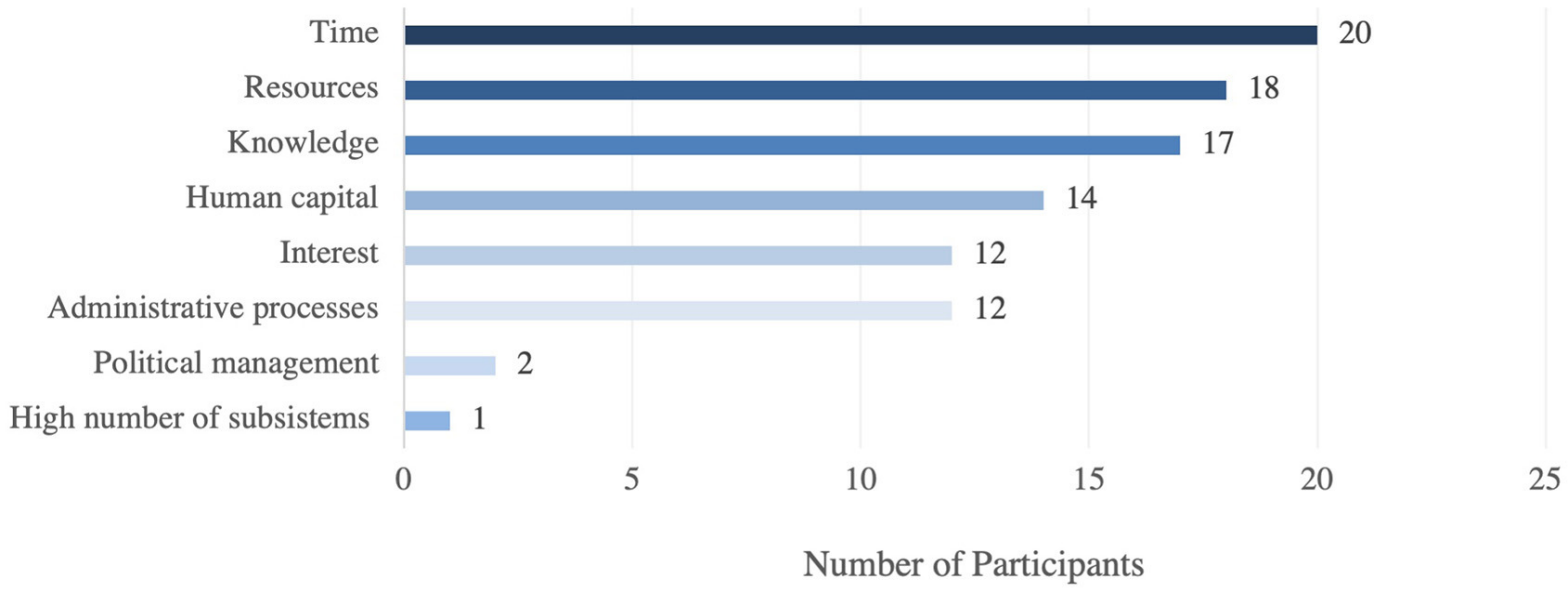
Main challenges in implementing systems thinking in the Region of the Americas.

More than half of the participants reported having had some challenges when implementing Systems Thinking. The main challenges of implementing systems thinking are related to time, resources, and knowledge (Figure 3).

Finally, the main benefits of applying systems thinking reported in the survey were in the articulation of a problem or need, helping with decision taking, and during coordination.

In addition, through the survey, 4 systems thinking groups/initiatives, 14 health strengthening initiatives, 7 programs/courses/trainings, 7 additional key stakeholders, and 5 publications in the Region of the Americas were identified.

### 3.3. Stakeholder mapping and prioritization

**Figure 4:**
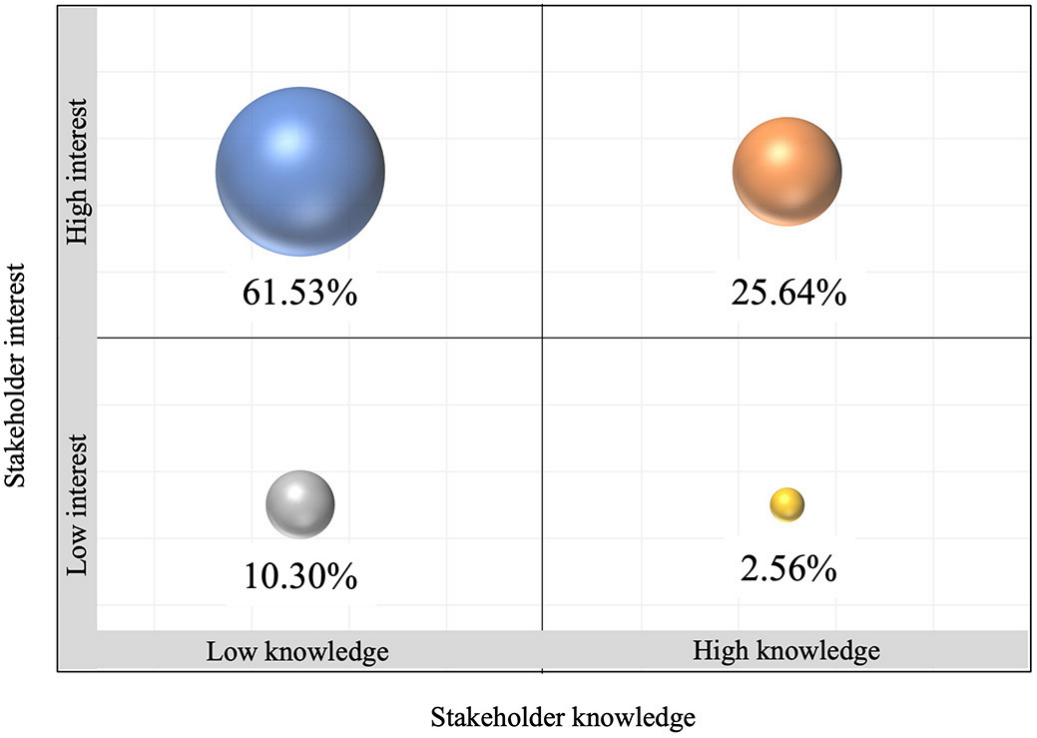
Levels of interest and knowledge about systems thinking among the participants in the survey.

The survey participants were classified and mapped according to their levels of interest and knowledge regarding systems thinking, as shown in Figure 4.

A 61.53% of participants described a low knowledge in systems thinking but high interest in developing capabilities and joining a community of interest. The 25.64% described both a high interest and high knowledge in the topic. Both this groups were identified as key segments, the first as individuals who will benefit from joining SYSTAC to gain capabilities in Systems Thinking. The second as key participators, instrumental in enhancing and sharing their experience regarding Systems Thinking in the region with the other stakeholders. Finally, the 2.56% of participants with high knowledge but low interest were classified as stakeholders to keep satisfied as they are influential in the field although minimally engaged (Figure 4).

In this stakeholder mapping exercise, it was not possible to identify the relationships between actors, their interests, and resources to understand linkages, relationships, interactions, and interdependencies among the components of a system that give rise to the observed behavior. Part of the differences for this detection is the need to have scopes by countries that allow more knowledge of local dynamics and realities to move toward a regional perspective according to levels of interest and influence in the global community of systems thinkers. Additionally, of the participating stakeholders 45% classified themselves as Managers, 33% as Researchers, 13% as Decision Makers and 10% as Practitioners. These categories were not mutually exclusive because people are not one-dimensional. Most of the stakeholder's when self-classifying identified themselves in the three categories. This added complexity to the mapping and classifying of their actions, application according to its role of systems thinking, and determination off significant relationships with other actors and stakeholders. For this reason, and due to a small sample size of participants in the survey we recognize that further work is needed to better understand the stakeholder Systems Thinking network in the region.

### 3.4. Workshop

The workshop was held on May 27 of 2021, 23 stakeholders participated, including the research team. There were representatives of multilateral organizations such as the Pan American Health Organization, institutions such as the Ministry of Health, and the Costa Rican Social Security Fund, local and regional academic institutions (universities and research centers), non-governmental organizations and the private sector. This allowed contributions to be made from different levels of operation, locally, regionally, and globally.

As mentioned above, the Systems Thinking in health definition outlined at the beginning of the results section was expanded on and validated with the workshop participants. Collective discussion and reflection through the case analysis made it possible to identify that the application of systems thinking in health interventions requires an approach focused on individuals, families, and communities, which recognizes and reduces the distance between the elements that make up the Health System and incorporates cultural and gender diversity, while at the same time harmonizing with other complex approaches such as Social Determinants and Health in All Policies.

Systems Thinking was conceived in the workshop as an approach that recognizes both organizational and civil society capacities, with multidisciplinary and inter-institutional cooperation being essential for strengthening teamwork. In addition, it was identified that continuous training for the development of capacities in the application of systems thinking in health implies continuous training processes that favor the application of its tools. Participants mentioned that it should be built from the bottom up with the active participation of civil society in the different stages, from design and planning to implementation, monitoring, and evaluation.

Despite the above, in practice, participants identified that systems thinking in the region faces different challenges, such as resistance to change and to the transformation of health processes. It was highlighted that barriers at the institutional level and different administrative disincentives hinder its application. Furthermore, institutional transparency, political will, and the articulation of the actors, are key to the successful application of Systems Thinking in health in the region.

It was identified that applying Systems Thinking brings different opportunities when designing, planning, executing, monitoring and/or evaluating health initiatives in the Region of the Americas, as it allows democratic and horizontal structures, while favoring empowerment for participation, integration, and synergy of all components of the Health System, continuous information flows and evidence generation. Thus, by conceiving health as a multidimensional and multifactorial element, the application of systems thinking allows the analysis and evaluation of all its dimensions, facilitating the integration between public and private sectors, integrating social, economic, and political aspects which in turn allow the expansion of perceptions and health scenarios.

## 4. Discussion

**Figure 5:**
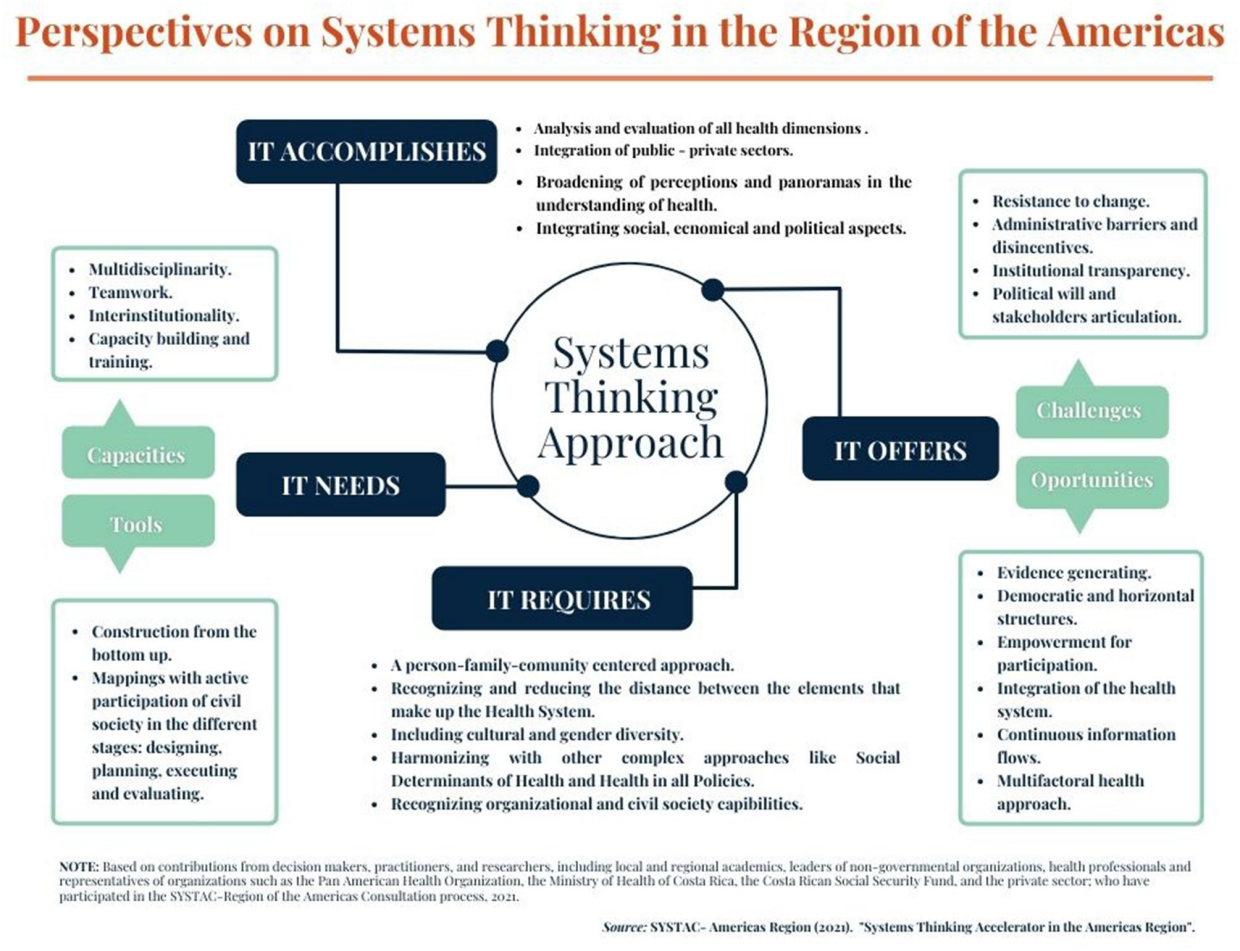
Perspectives on systems thinking in the Region of the Americas.

The main takeaways from the needs assessment survey, stakeholder mapping and prioritization, and the workshop were integrated to better understand the challenges and opportunities to accelerate Systems Thinking within the region. These integrated findings are presented in Figure 5 and discussed below.

### 4.1. Do the capabilities and interest to apply Systems Thinking in the Region of the Americas exist?

Participants show great interest in developing and applying the tools of systems thinking, although they report little theoretical knowledge in this regard. This research identified that only 28% of participants reported a high knowledge of the tools and approaches of system thinking, although 87% reported a high interest in developing skills related to Systems Thinking and its application in health. Having said this, stakeholders may already use Systems Thinking tools in their day-to-day jobs without identifying it as such. Although 31.4% of survey participants reported not having used Systems Thinking tools, all these same participants later selected one or more tools from a list, as tools that they use when preforming their work. These findings highlight that there is an interest in Systems Thinking, and an opportunity to build recognition and capabilities around this methodology. Furthermore, the research team believes that developing these skills and capabilities through a community such as SYSTAC would have a positive impact in the Health Sector in the Region of the Americas within research, project implementation, project evaluation and design.

### 4.2. What are the main challenges and opportunities to applying systems thinking, in health, in the region of the Americas?

In practice, participants identified that applying system thinking faces different challenges, such as:

- Resistance to change and to the transformation of health processes.
- Barriers at the institutional level and different administrative disincentives such as lack of transparency and inter and intra-institutional collaboration.
- Difficult stakeholder integration.
- Low political will to implement systems thinking due to time constraints,
- Lack of resources and lack of funding.
- Gaps in knowledge.

Some of which (e.g., access to resources, training to develop capabilities, reducing resistance to change through education, etc.), could be alleviated, or minimized through initiatives such as SYSTAC. Furthermore, a Systems Thinking community could help build political will and be instrumental in the articulation of the actors, which are key to the successful application of Systems Thinking, in health, in the region. Additionally, such a community represents a valuable learning and knowledge exchange opportunity for systems thinkers.

Although survey participants described using Systems Thinking during research, more than half also mentioned using it in practice, when implementing and evaluating projects, which hints that the use of Systems Thinking goes beyond the purview of research. Additionally, study results indicate that there is a clear benefit to strengthening and building personal and institutional capacities in systems thinking theory and practice, in the region. During the discussion and reflection of the case analysis in the workshop, it was identified that the application of systems thinking in health interventions helps frame the interventions from additional perspectives to the providers' perspective, such as that offered by the individuals/patients, families, and communities, integrating cultural and gender diversity, which in turn can have a positive impact on the health system. Furthermore, systems thinking was described as a multidisciplinary and inter-institutional approach which strengthens teamwork, is instrumental for gathering evidence, creating democratic and horizontal structures, empowers the different stakeholders and sectors in health systems to participate and collaborate, and promotes a continuous flow of information.

### 4.3. Proposed next steps for the acceleration of systems thinking in health in the region of the Americas

As a result of the challenges and opportunities, the research team identified the importance of designing and prioritizing a consensual course of action or roadmap for the acceleration of system thinking in health in the Region of the Americas through a SYSTAC community. This interest community should be built from the bottom up with the active participation of civil society in the different stages, from design and planning to implementation, monitoring, and evaluation.

As a first step, it is necessary to expand the call to include a larger number of stakeholders, decision-makers, practitioners, and researchers in the region. Although the process identified a high level of interest in learning about systems thinking and its tools, the number of stakeholders who responded was limited, in part due to the COVID pandemic taking place at the same time as the study. In addition, expanding the type of stakeholders involved would promote inclusivity and ownership, furthering the goal of building System Thinking capabilities in the region. This recommendation will be a priority starting point in the continuity of the process toward community building. Furthermore, it is also necessary to expand the study to cover the multiplicity of tools within System Thinking such as those related to change theory, among many others.

Moreover, further analysis is required to (a) document/describe systems thinkers' networks to be able to strengthen them by linking key stakeholders (existing and new) and ideas, and (b) build a more in-depth understanding of the capacity needs (e.g., such as a more comprehensive understanding of the existing capabilities for the multiplicity of System Thinking tools not only the ones explored in this study) for which training opportunities are required.

Some initial thoughts on possible initiatives toward capacity building are:

- Creating a platform that allows to disseminate existing information and resources on systems thinking, providing access to a repository of resources from which to learn about the main elements and tools of systems thinking.
- Promoting continuous education, and apply a train-the-trainer strategy, by training educators who work in public health schools and other schools which prepare stakeholders who then go into the health system, so that they pass on the knowledge to their students.
- As there are currently some training resources on systems thinking in health in the region, an effort to make them accessible to practitioners, managers, researchers, and decision-makers, should be undertaken.

Having the support of SYSTAC and partner organizations in the different regions in the development and implementation of these initiatives would allow the leveraging of solid and existing structures as a reference point, which would be instrumental in the acceleration of the application of systems thinking in the region.

## Data availability statement

The raw data supporting the conclusions of this article will be made available by the authors, without undue reservation.

## Author contributions

All authors listed have made a substantial, direct, and intellectual contribution to the work and approved it for publication.
