# Supplementary material for: Accelerating systems thinking in health: Perspectives from the region of the Americas
Source: Front Public Health. 2023 Mar 16;11:968357. doi: 10.3389/fpubh.2023.968357 (PMC10060521; doi:10.3389/fpubh.2023.968357)
Supplement: Supplementary file 1 [file Data_Sheet_1.docx]

**PURPOSE:**

With the purpose of exemplifying the conceptual and practical application of Systems Thinking and its tools in health initiatives, the Costa Rican experience of the Navigation Project for Breast Cancer Patients "Women who Save Lives" is presented, which is developed in national, regional and specialized hospitals of the Costa Rican Social Security Fund.

**CONTEXT:**

According to data from the Costa Rican Social Security Fund (CCSS), in 2010 breast cancer in the country reached the second place by incidence with a rate of 44% and the first by mortality with a rate of 13%, being a priority situation in health. ^[[1]](#footnote-1)^According to the study entitled *Epidemiological and socio-institutional perspective of breast cancer in Costa Rica*, ^[[2]](#footnote-2)^ for this date there was still no relevant information on clinical aspects, treatment, active follow-up, times and quality of care, management and impact, or on the real costs of breast cancer care.

Althoughby this time there was already the National Cancer Network within the public health services, it was necessary to improve the diagnosis, evaluation and coverage of mammograms as a screening mechanism, long waiting lists were counted and there were no programs with social participation, campaigns or consolidated and permanent strategies that analyzed other social determinants in the comprehensive approach to breast cancer and / or promoted healthy lifestyles in the national, communal or individual context. Despite this, it was identified the existence of 14 non-governmental organizations (NGOs) that provided, among others, accompaniment services, as well as education and prevention activities; but without articulation and with duplication of their activities.

The Patient Navigation Project (PNP) began in 2012 in response to the findings on the barriers to the care of this pathology from the perspective of patients, health personnel, as well as from the non-governmental organizations that support them, being a CONARE extension project led by the School of Public Health-University of Costa Rica (UCR) and the Center for General Studies-National University (UNA^[[3]](#footnote-3)^). in linking the NGOs: Asociación Resurgir, APROD-PA, Voluntarios de Bernini and the Patients-Roche Project in the San Vicente de Paul hospitals in Heredia, San Rafael de Alajuela and Hospital de las Mujeres. By 2016, it was expanded to the health services of the Dr. Rafael Ángel Calderón Guardia Hospital, San Juan de Dios Hospital, Hospital México, and San Vicente de Paul Hospital in Heredia, and eight other NGOs joined.

**DESIGN AND EXECUTION OF THE NPN:**

The PNP links civil society, academia and health services, each sector having an interdependent role of utmost relevance for the comprehensive approach focused on the timely resolution of the needs felt and expressed by the users, with the participation of the navigators in all stages of the model, from design and execution to its monitoring and evaluation, developing participatory sessions where the contributions of women who have experienced this disease have been the main resource, and making use of various management tools, planning, among others.

Its current work dynamics incorporate as a priority the training of the volunteers of the participating NGOs, who are mostly women survivors of the disease, so that they can influence the care process as key informant pairs (counting the process) and as support (accompanying the process) in scenarios such as the breast medical session, This implies a model of integration of civil society into the health system. Once trained, the navigators participate in the entire care process, carry out recruitment, accompaniment and follow-up of patients, support the identification, resolution of barriers and management of inequities by identifying bottlenecks from the voice of the users, being recognized by peers and transferred to health services, for the reduction of waiting times, early diagnosis and timely treatment. All the information provided by the users to the volunteers is deposited in an exclusive database of the project that allows identifying unattended medical and non-medical needs and, with this, generating scientific evidence for decision making.

The Costa Rican Social Security Fund assumes a fundamental role, because after the assignment of a person as a "liaison" for the follow-up of the project in the services, it makes the necessary transformations to reduce and resolve those situations that afflict patients and that have been consolidated as barriers in their care processes. In addition, it facilitates the access of volunteers to health services, supports their training and provides operational information, user data and collaborates with the logistics of activities for patients and other actions of relevance to the PNP.

For its part, the School of Public Health is responsible for the technical coordination, project management and data analysis from the Social Determinants that affect the breast cancer care process, deepeningthe barriers to access to health services that users may encounter. The final result is the generation of scientific evidence from the information provided by users to navigators, to support the reorientation of health services and decision-making.

**RESULTS OF THE NPP:**

To date, there is important information about its results and impacts on people and services, with 4162 women receivingsupport from women navigators. This has allowed the generation of the following evidence: more than 80% of the patients who **attended the breast session have been recruited**, more than **75% of patients recruited before starting their treatment and the remaining 25% before their surgery**, who have also benefited from **10462 face-to-face or virtual accompaniments and 45051 effective follow-ups**, being relevant that today there are **more than1962 cases in which users have been the ones who come to the project in request for support.**

Innovation indicators have also been proposed: accessibility, adoption, appropriation, loyalty, cost, coverage and staggering of them. Interms of ***Accessibility***, it is estimated that 95% of the users of the participating hospitals are covered globally, with 81 active navigators as of June 2020 that provide voluntary support, for the Adoption indicator it is necessary that 51% of the support in the resolution of barriers is carried out by specialist doctors and 29% other treating services, In addition, it participates in the breast sessions of three of the hospitals that develop the project. On the ***Appropriation***, 7 of 8 NGOs that started the project remain linked which has allowed 100% of the fulfillment of the schedule, exceeding the expectation of coverage of the project between 28% and 40%, in addition if the Costs are contemplated, with the resolution of barriers an overall reduction of 136 days in the care process was evidenced in 3 participating hospitals, which has allowed us to recognize that, the greater the coverage, the greater the reduction in waiting times.

1. Ministry of Health. (May 26, 2021). *Cancer Statistics – National Tumor Registry.* https://www.ministeriodesalud.go.cr/index.php/vigilancia-de-la-salud/estadisticas-y-bases-de-datos/estadisticas/estadistica-de-cancer-registro-nacional-tumores [↑](#footnote-ref-1)
2. Sáenz, M.R. (et al). (2011). *Epidemiological and socio-institutional perspective of breast cancer in Costa Rica.* (1st ed.), Heredia, UNA. [↑](#footnote-ref-2)
3. DITTO [↑](#footnote-ref-3)
